# Analysis of the role of *BrRPP1* gene in Chinese cabbage infected by *Plasmodiophora brassicae*

**DOI:** 10.3389/fpls.2023.1082395

**Published:** 2023-01-25

**Authors:** Wenjie Ge, Mingcan Lv, Hui Feng, Xinlei Wang, Bo Zhang, Ken Li, Jing Zhang, Jiawei Zou, Ruiqin Ji

**Affiliations:** Liaoning Key Laboratory of Genetics and Breeding for Cruciferous Vegetable Crops, College of Horticulture, Shenyang Agricultural University, Shenyang, Liaoning, China

**Keywords:** clubroot disease, Chinese cabbage, *BrRPP1*, *Plasmodiophora brassicae*, resistance gene

## Abstract

**Introduction:**

The clubroot disease caused by *Plasmodiophora brassicae* (*P. brassicae*) poses a serious threat to the economic value of cruciferous crops, which is a serious problem to be solved worldwide. Some resistance genes to clubroot disease in *Brassica rapa* L. ssp *pekinensis* cause by *P. brassicae* have been located on different chromosomes. Among them, *Rcr1* and *Rcr2* were mapped to the common candidate gene *Bra019410*, but its resistance mechanism is not clear yet.

**Methods:**

In this experiment, the differences of *BrRPP1* between the resistant and susceptible material of Chinese cabbage were analyzed by gene cloning and qRT-PCR. The gene function was verified by Arabidopsis homologous mutants. The expression site of *BrRPP1* gene in cells was analyzed by subcellular localization. Finally, the candidate interaction protein of *BrRPP1* was screened by yeast two-hybrid library.

**Results:**

The results showed that the cDNA sequence, upstream promoter sequence and expression level of *BrRPP1* were quite different between the resistant and susceptible material. The resistance investigation found that the Arabidopsis mutant *rpp1* was more susceptible to clubroot disease than the wild type, which suggested that the deletion of rpp1 reduces resistance of plant to clubroot disease. Subcellular location analysis confirmed that *BrRPP1* was located in the nucleus. The interaction proteins of *BrRPP1* screened from cDNA Yeast Library by yeast two-hybrid are mainly related to photosynthesis, cell wall modification, jasmonic acid signal transduction and programmed cell death.

**Discussion:**

*BrRPP1* gene contains TIR-NBS-LRR domain and belongs to R gene. The cDNA and promoter sequence of BrRPP1 in resistant varieties was different from that in susceptible varieties led to the significant difference of the gene expression of *BrRPP1* between the resistant varieties and the susceptible varieties. The high expression of *BrRPP1* gene in resistant varieties enhanced the resistance of Chinese cabbage to *P. brassicae*, and the interaction proteins of *BrRPP1* are mainly related to photosynthesis, cell wall modification, jasmonic acid signal transduction and programmed cell death. These results provide important clues for understanding the mechanism of BrRPP1 in the resistance of B. rapa to *P. brassicae*.

## 1 Introduction

Chinese cabbage is a cruciferous *Brassica* crop with both nutritional value and economic value. However, Chinese cabbage is often harmed by *P. brassicae*, which leads to a soil-borne disease-clubroot disease, and brings huge losses to yield and economy (Ludwig-Müller and Schuller, 2008). The infection cycle of *P. brassicae* was mainly divided into two stages, infection stage (root hair infected) and disease stage (hypocotyls, cortex and stele infected) (Hirai, 2006). At the later stage of the disease, the overground part of the host plant shows the phenomenon of yellowing and wilting leaves, and the underground part forms swollen nodules, which will affect the root function and reduce the host’s the absorption of water and nutrients (Wang et al., 2016).

So far, there is no effective strategy to control clubroot disease of Chinese cabbage (Diederichsen et al., 2009). Therefore, it has become an important research direction to find more disease resistance genes and reveal their resistant mechanism. In nature, plants are often invaded by various pathogens. Cell wall is the first cell barrier against pressure, which is composed of polysaccharide skeleton, protein and polymer (Lampugnani et al., 2018). Plants can resist a large number of pathogens through the waxy layer and stratum corneum of the cell wall and outer epidermis (Houston et al., 2016). In plants, the key steps of photosynthesis and synthesis of defense related hormones occur in chloroplasts (Lu et al., 2018). In addition, chloroplast is the main production site of reactive oxygen species and nitric oxide, and the site of calcium signal transduction. Photosynthesis can also be affected by defense related hormones and signaling molecules (Fischer et al., 1986; Seemann and Sharkey, 1987; Popova et al., 1996).

When infected by pathogens, the biological stress induced by pathogens will trigger complex signal cascade responses regulated by hormones. The stress and defense related hormone jasmonic acid (JA) is considered as one of the basic components of the pathogen induced response (Kazan and Lyons, 2014). JA defense response is necessary for defense against biotic stress and abiotic stress, and works cooperatively with other hormones (Wasternack, 2007; Wasternack and Hause, 2013). Hormone dependent pathways lead to the expression of defense related genes and the production of antimicrobial secondary metabolites (Glazebrook, 2001).

In recent years, many resistance genes to clubroot disease have been located in Chinese cabbage including *Crr1*, *Crr2* (Suwabe et al., 2003), *Crr3* (Saito et al., 2006), *Crr4* (Suwabe, 2006), *CRa* (Matsumoto et al., 1998), *CRb* (Kato et al., 2013), *CRc* (Sakamoto et al., 2008), *CRk* (Suwabe et al., 2003), *Rcr1* (Chu et al., 2014), *Rcr2* (Huang et al., 2017), *PbBa3.1*, *PbBa3.2* (Chen et al., 2013). It is worth noting that *Bra019410* has also been mapped as a candidate resistant (*R*) gene for many times in the research of Chinese cabbage resistance to *P. brassicae* infection. For example, Yu et al. (2016) mapped *Bra019409* and *Bra019410* as candidate genes for the target region of *Rcr1* by the combined technology of BSA and KASP. *Rcr2* was also fine-located between two SNP sites by the combined technology of BSA and KASP, and finally *Bra019410* and *Bra019413* were located as the candidate genes of *Rcr2* (Huang et al., 2017). In summary, *Rcr1* and *Rcr2* share a common candidate gene *Bra019410*. This suggested that *Bra019410* should be a key resistant gene in *Brassica rapa* resisted to clubroot disease.

Our research found *Bra019410* is homologous to the *Arabidopsis RPP1* (Recognition of *Peronospora parasitica*1), which is a *R* gene and encodes the TIR-NBS-LRR protein, so we named it as *BrRPP1*. *RPP1* is also considered as a candidate gene when crops respond to pathogens. For example, *RPP1* plays a key role in the resistance of *Brassica napus* to blackleg disease (caused by the fungal pathogen *Leptosphaeria maculans*) (Larkan et al., 2014) and *Brassica juncea* resistance to *Sclerotinia sclerotiorum* (Rana et al., 2019).

Pathogen ‘recognition’ is considered as the first step of disease resistance, as a signal of general defense response (Botella, 1998). Most *R* genes in plants belong to NBS-LRR (Nucleotide binding sites rich in leucine repeats) family genes. The multi-domain structure of the NBS-LRR family gene enables it to have multiple functions and is responsible for pathogen identification and signal transduction (Takken et al., 2006). As a *R* gene, *RPP1*, participates in host-pathogen interaction and is confirmed to be a downy mildew resistant gene (Holub and Beynon, 1997; Botella, 1998). RPP1 can bind to ATR1 and form a tetramer through the oligomeric interface of the NBS domain. This tetramer has enzymatic activity and can catalyze the hydrolysis of NAD^+^ and activate downstream EDS1 proteins such as (enhance disease susceptibility 1) and NRG1 (N requirement gene 1) can regulate the death response of cells (Wan et al., 2019).

Although *BrRPP1* has been mapped as a candidate gene for resistance to clubroot disease in Chinese cabbage, its resistance function and its resistance mechanism to clubroot is still not clear. In this study, the sequence difference of *BrRPP1* between resistant and susceptible material of Chinese cabbage was found, and its interaction proteins were obtained and analyzed, which can provide important information for the study of mechanism of *BrRPP1* in the resistance of Chinese cabbage to *P. brassicae*.

## 2 Materials and methods

### 2.1 Materials

The materials used in the experiment were resistant variety ‘SN205’ and susceptible variety ‘SN742’ of Chinese cabbage, physiological race *no.4* of *P. brassicae*, Colombian wild-type ‘WT’ of *Arabidopsis thaliana* and tobacco ‘*Nicotiana Benthamiana*’. All the above experimental materials were preserved by the Vegetable Genetics and Breeding Laboratory of Shenyang Agricultural University. The *Arabidopsis* mutant *rpp1* (‘*SALK_065253*’) was purchased from ABRC (https://abrc.osu.edu/).

### 2.2 Acquisition of *BrRPP1*, and analysis of the difference *BrRPP1* between resistance and susceptible Chinese cabbage

The reference sequence of *BrRPP1* was obtained by searching from *Brassica* database (BRAD (brassicadb.cn)). Then the sequence validity was checked by Nucleic acid BLAST (http://www.ncbi.nlm.nih.gov/blast/) in the National Center of Biotechnology Information (NCBI). The gene conservative structure domain was predicted by NCBI protein conserved domain database (https://www.ncbi.nlm.nih.gov/Structure/cdd/wrpsb.cgi).

To obtain accurate cDNA sequences of *BrRPP1* from the ‘SN205’ and ‘SN742’, three segmented primers (*BrRPP1*-1, *BrRPP1*-2 and *BrRPP1*-3) with *Eco*3II sites were designed using Primer 5 software (**Table 1**). The three corrected amplified fragments from the cDNA of ‘SN205’ or ‘SN742’ were digested by *Eco*3II (Beijing NEB, China) respectively, and then ligated to pBWA(V)BS vector linearized by *Eco*3II using T4 ligase (Tiangen, Beijing, China). The connecting products pBWA(V)BS-SN205-*BrRPP1* and pBWA(V)BS-SN742-*BrRPP1* were transformed into the competent cells of Trans10 *E. coli* respectively. The correctness of recombinant clones were detected with primers (F: cagtGGTCCacacatgatttcgatcgattttttgaaaaaaaaa and R: cagtgGTCTCatacataacatgaggaggaggaggtttc) and the colony with the correct band position was sequenced (Sangon Inc, Shanghai, China). The three-dimensional structure of BrRPP1 protein in ‘SN205’ and ‘SN742’ was predicted using SWISS-MODEL online tool (https://swissmodel.expasy.org/interactive).

**Table 1:** The Primers for amplifying the full cDNA sequence of *BrRPP1.*

Primer Name	Primer sequence (5’-3’)	Begin-end (bp)
*BrRPP1*-1	F: cagtGGTCTCacaacatgagatttcgatcgtttttgaaa	1-1411
	R: cagtGGTCTCaatcctataacctttagtcccaaag	
*BrRPP1*-2	F: cagtGGTCTCaggatcttatttcgaaggaaggc	1407-3060
	R: cagtGGTCTCttgcttcttgattcagtttaaagc	
*BrRPP1*-3	F: cagtGGTCTCtcgtgaacttgtaatcaagggatgcacga	2855-3471
	R: cagtGGTCTCatacattaacatgagggagccaaggtttc	

### 2.3 Cloning and element analysis of the promoter of *BrRPP1*

Promoter primers (*BrRPP1pro*-F: CTGTTCTCGTATCCTTCAC and *BrRPP1pro*-R: CTCATCTGCTTTCTCTTTTTC) were designed according the upstream 2 kb sequence of *BrRPP1* from *Brassica* database (brassicadb.cn). The DNA of ‘SN205’ and ‘SN742’ were used as templates respectively to amplify the promoter sequences of *BrRPP1*. Promoter elements were analyzed using online software PlantCARE (http://bioinformatics.psb.ugent.be/webtools/PlantCARE/htmL/).

### 2.4 Quantitative real-time reverse transcription PCR analysis

cDNA was obtained from ‘SN742’ roots on the 14^th^ day after inoculation of *P. brassicae* (at this time, *P. brassicae* spores were found in root hairs under the compound microscope) and on the 42^nd^ day after inoculation (at this time, obvious swelling appeared in the roots), respectively. In addition, the roots cDNA of uninoculated ‘SN742’ and ‘SN205’ at above same stage was used as control and then to analyze the difference of *BrRPP1* expression between resistance and susceptible materials. Using Quantstudio6 (ThermoFisher, Waltham, MA, USA) and the fluorescence quantification kit UltraSYBR Mixture (Low ROX, Cwbio, Jiaosu, China), we analyzed the expression pattern of *BrRPP1* in the roots of Chinese cabbage at two sampling stages after infection by *P. brassicae* by qRT-PCR. The specific primers were designed, and the *BrActin* gene (Gao et al., 2020) was used as the internal control (primers are shown in **Table S1**). The relative expression of *BrRPP1* was analyzed using the 2^−ΔΔCt^ method (Livak and Schmittgen, 2001) and SigmaPlot 12.5 (Systat Software, Inc., San Jose, CA, USA). Three replications were performed for each different treatment. The difference significance test was analyzed using the program SPSS v.26 (IBM, Armonk, NY).

### 2.5 Disease-resistance identification of *Arabidopsis thaliana* mutant *rpp1* (*SALK_065253*)

Seeds of *Arabidopsis* mutant *rpp1* and wild-type ‘WT’ were vernalized at 4 °C low-temperature for 3-5 days and then evenly sowed in nutritious bowl. When seedlings grew to 10 true leaves, DNAs of *rpp1* and ‘WT’ leaves were extracted by modified CTAB method (Springer, 2010). The ‘three-primer’ PCR was performed to select the homozygous mutants. The detection primers (LP: ACCATTCATTGTTCCTTGCAG; RP: ATGATAATGAAGGACCCCTCC; LB: ATTTTGCCGATTTCGGAAC) were obtained from the SIGnAL website (http://signal.salk.edu/tdnaprimers.2.htmL). The homozygous mutants were further cultured until offsprings were obtain.

The *P. brassicae* suspension with a concentration of 10^7^/mL was prepared according to Lv et al. (2021). The seeds of the *rpp1* and ‘WT’ were sterilized and seeded in a petri dish containing moist filter paper to accelerate germination at 25 °C. After about 24 hours in darkness, the culture dishes were transfered into a light incubator at 25 °C, with 16 hours of light and 60% humidity. When two cotyledons were extended, *P. brassicae* suspension were sprayed on the roots of mutant *rpp1* and ‘WT’ for inoculation. Five randomly selected plants were observed at 24 h intervals. The roots of seedlings were cut into 1 cm sections, dyed with 1% toluidine blue for 15 min, and decolorized with sterile water for 5 min, and then observed under a composite microscope (Eclipse 80i; Nikon, Tokyo, Japan).

### 2.6 The subcellular localization analysis of *BrRPP1*

To construct the 35S::pGPTVII.GFP-*BrRPP1* expression vector, the *BrRPP1* coding sequence with the termination codon removed was amplified from pBWA(V)BS-SN205-*BrRPP1* using BrRPP1-GFP-F:CGCCACTAGTGGATCCatgagatttcgatcgtttttgaaaga; BrRPP1-GFP -R:GAGCGGTACCCTCGAGacatgagggagccaaggtttcc, and inserted into the pGPTVII.GFP vector using NovoRec homologous recombinase (Novprotein, Shanghai, China).

The plasmid of 35S::pGPTVII.GFP*-BrRPP1* and 35S::pGPTVII.GFP (as a control) was introduced into *Agrobacterium* strain GV3101 respectively. The strains were injected into 4-week-old tobacco leaves as described previously (Sainsbury et al., 2009). After 48 hours in darkness, the tobacco leaves were stained with 4,6-diamidino-2-phenylindole (DAPI, Coolaber, Beijing, China), and observed under a Confocal laser microscope (TCS SP8-SE, Leica, Wetzlar, Germany).

### 2.7 Interaction proteins screening of BrRPP1 from yeast two-hybrid library

To construct pGBKT7-BrRPP1 bait vector, the *BrRPP1* coding sequence with the termination codon removed was amplified from pBWA(V)BS-SN205-*BrRPP1* using *BrRPP1*-BD-F: CGCACTAGTGGATCCatgagatttcgatcgtttttgaaaga and *BrRPP1*-BD-R: AAGGAAAAAAGCGGCCGCacatgagggagccaaggtttcc, and the products and PGBKT7-T7 were respectively digested with *Bam*HI and *Not*I (NEB, Beijing, China) and connected. The obtained pGBKT7-*BrRPP1* bait vector was transferred into the freshly prepared competent cells of Y2H yeast. The self-activation and toxicity of positive colonies and yeast library screening was carried out by mating hybridization according to Xu et al. (2020). Functional annotation of candidate interaction proteins of BrRPP1 obtained from the screening library were analyzed by NCBI and *Brassica* database, and the expression pattern of those proteins with high detection rate was analyzed by qRT-PCR (primers are shown in **Table S1**).

## 3 Results

### 3.1 Acquisition and analysis of *BrRPP1* gene sequence

**Figure 1:**
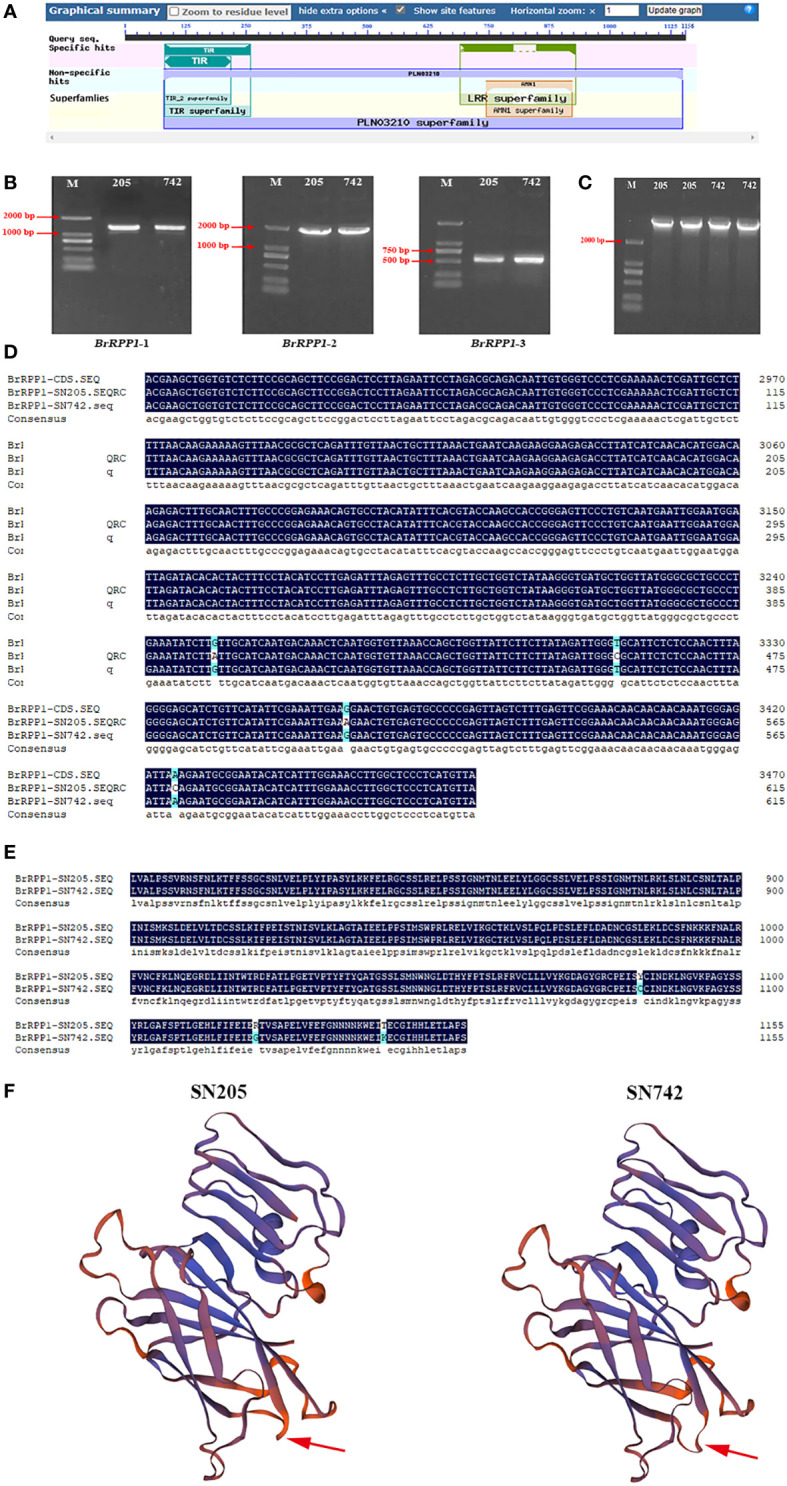
Acquisition and analysis of *BrRPP1* gene sequence. **(A)** Conservative domain prediction of *BrRPP1*. **(B)** *BrRPP1* fragment amplified from ‘SN205’ and ‘SN742’ with three segmented primers *BrRPP1-1, BrRPP1-2 and BrRPP1-3*. **(C)** The cDNA full-length of *BrRPP1* in ‘SN205’ and ‘SN742’. **(D)** cDNA sequence alignment of *BrRPP1* in different regions between ‘SN205’ and ‘SN742’. **(E)** Amino acid sequences alignment of BrRPP1 in different regions between ‘SN205’ and ‘SN742’. **(F)** Protein structure prediction of BrRPP1 in ‘SN205’ and ‘SN742’. The arrow points to the different positions.

The reference sequence of *BrRPP1* gene (*Bra019410*) obtained from *Brassica* database contains an open reading frame (ORF) with a length of 3471 bp, which is located on chromosome A03. The accession number of *Bra019410* in the NCBI database is XM_009138983.3. The conservative structure domain prediction of *BrRPP1* showed that there are a TIR conserved domain in the region of 85-259 aa, and a LRR conserved domain in the region of 690-929 aa (**Figure 1A**). Therefore, *BrRPP1* is a TIR-NBS-LRR-like protein.

The lengths of products amplified by *BrRPP1*-1, *BrRPP1*-2 and *BrRPP1*-3 were 1141 bp, 1654 bp and 617 bp, respectively, and the results were consistent in ‘SN205’ and ‘SN742’ (**Figure 1B**). The ligation products of the three cDNA fragments were both 3471 bp in ‘SN205’ and ‘SN742’ (**Figure 1C**). Comparing the cDNA sequences of *BrRPP1* from ‘SN205’ and ‘SN742’ with the reference genome, it was found that the sequence in ‘SN742’ is consistent with the reference sequence. However, it is cDNA different between ‘SN205’ and reference sequence, with four single nucleotide polymorphisms (SNPs) (G to A, T to C, G to A and A to C) in the 3450-3471 bp region (**Figure 1D**). The change of base leads to the change of amino acid sequence and three-dimensional structure of BrRPP1 protein (**Figures 1E, F**).

### 3.2 Difference analysis of promoter sequence of *BrRPP1* between resistance and susceptible Chinese cabbage

**Figure 2:**
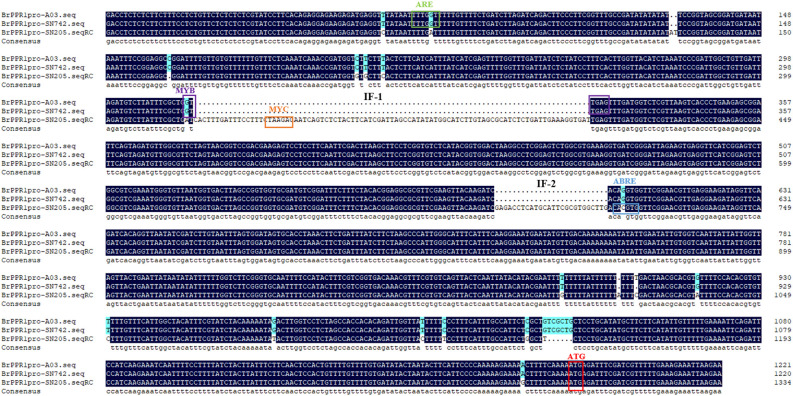
Promoter sequence and element analysis of *BrRPP1* in resistance and susceptible materials of Chinese cabbage.

The upstream 1167 bp promoter region of coding sequence of *BrRPP1* were cloned from ‘SN205’ and ‘SN742’. Sequences alignment found that there are two additional insert fragments (IF-1, IF-2) and multiple SNPs in the promoter region of *BrRPP1* in the ‘SN205’ compared with in the ‘SN742’. The prediction of promoter elements found that the *BrRPP1* promoter region amplified form ‘SN742’ or ‘SN205’ contains basic elements, light-responsive elements and corresponding elements of biotic and abiotic stress (**Table 2**). However, the number of cis-acting elements was different in the two materials. Analyzing the difference of biotic and abiotic stress elements in *BrRPP1* promoter between ‘SN205’ and ‘SN742’, it was found that there was a MYC transcription factor binding site in one of inserted segments (IF-1) in *BrRPP1* promoter of ‘SN205’, and a MYB binding site was truncated by the inserted segment IF-1. In addition, compared with *BrRPP1* promoter of ‘SN742’, an ARE element was missing and an ARBE element was adding in that of ‘SN205’ (**Figure 2**).

**Table 2:** Promoter element analysis of *BrRPP1* in resistance and susceptible materials of Chinese cabbage.

Type	Regulated element	Biological function	Amount	
			SN205	SN742
Basic element	CAAT-box	Promoter and enhancer regulatory elements	5	5
	TATA-box	Transcription start site	7	9
Light-responsive element	ACE	Components involved in light response	1	0
	AE-box		1	1
	G-Box		3	4
	GT1-motif		1	1
Corresponding elements of biotic and abiotic stress	ABRE	Abscisic acid response element	3	2
	ARE	Antioxidant response element	1	2
	MYB	transcription factor binding site	5	6
	MYC		2	1
	W-BOX		1	1

### 3.3 The expression pattern analysis of *BrRPP1*

**Figure 3:**
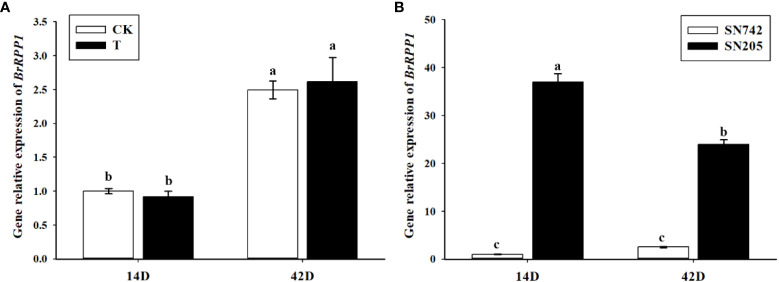
The analysis of relative expression of *BrRPP1* gene. **(A)** The expression difference of *BrRPP1* between uninoculated and inoculated roots with *P. brassicae.* 14D, the 14th day after inoculation of *P. brassicae*. 42D, the 42th day after inoculation of *P. brassicae*. CK, uninoculated control roots. T, roots treated with *P. brassicae*. **(B)** The expression of *BrRPP1* in roots of ‘SN742’ and ‘SN205’ uninoculated with *P. brassicae*. Data represent the mean ± standard deviation (n = 3); letters indicate significant differences at p ≤ 0.05, according to Duncan’s multiple range test.

In order to analyze the expression pattern of *BrRPP1* gene, qRT-PCR showed that there was no significant difference between the expression level of *BrRPP1* in ‘SN742’ roots of 14^th^ and 42^nd^ days after inoculation of *P. brassicae* and that in uninfected roots of Chinese cabbage (**Figure 3A**). However, the comparative analysis of the expression of *BrRPP1* between ‘SN742’ and ‘SN205’ showed that it was significantly higher in ‘SN205’ than in ‘SN742’ at both sampling dates (**Figure 3B**).

### 3.4 Identification of disease resistance of *Arabidopsis* mutant *rpp1*

**Figure 4:**
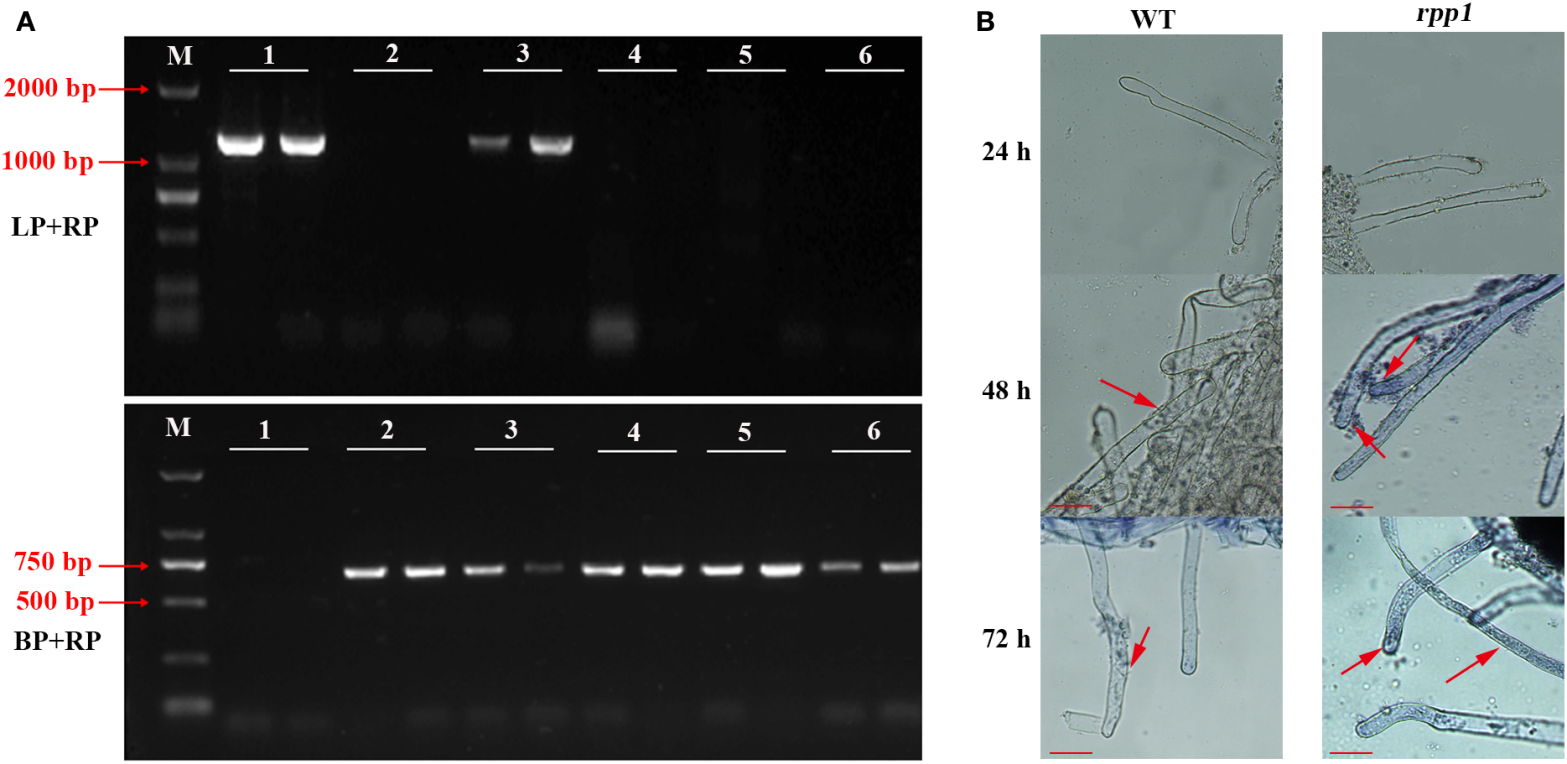
Disease resistance identification to *P. brassicae* in *Arabidopsis* mutant *rpp1*. **(A)** The identification results of *Arabidopsis* mutant *rpp1* by ‘three-primers’. M, D2000 bp marker; 1, wild type of *Arabidopsis* with two replicates; 2-6, different plants of *Arabidopsis* mutant *rpp1* with two replicates. **(B)** *P. brassicae* infection observation for wild-type (WT) and *Arabidopsis* mutant *rpp1*. The red arrow, the position of the spore or sporangium. Scale bar=50 μm.

Identification of *Arabidopsis* mutants by ‘three-primers’ showed that a fragment of about 1280 bp was amplified in No.1 and No.3 by LP+RP, and a fragment of about 720 bp was amplified in No.2, 3, 4, 5 and 6 by LB + RP (**Figure 4A**). In theory, LP and RP are located on both ends of T-DNA insertion site, and LB is located at T-DNA insertion site, the length of LP + RP amplification products should be 1280 bp, and the length of LB + RP amplification products should be 594-894 bp.

There was only a band of 1280 bp in No. 1, so it was a wild-type mutant without T-DNA insertion, while two bands of 1280 bp and 720 bp was amplified in No.3, so it was a heterozygous mutant with unilateral T-DNA insertion. There was only a band of 720 bp in No. 2, 4 and 6, so they were homozygous mutants.

After infection with *P. brassicae*, the roots of ‘*rpp1*’ and ‘WT’ were both not be infected within 24 h. After 48 h, primary spores appeared in the root hairs of the ‘*rpp1*’ mutants, while zoospores only attached to the surface of root hair of ‘WT’. After 72 h, the root hair of ‘*rpp1*’ was more severely infected by spores, and the primary spores appeared in the root hairs of ‘WT’ (**Figure 4B**). The results indicated that the absence of *RPP1* could accelerate the infection process of *P. brassicae*.

### 3.5 The subcellular localization analysis of BrPPR1

**Figure 5:**
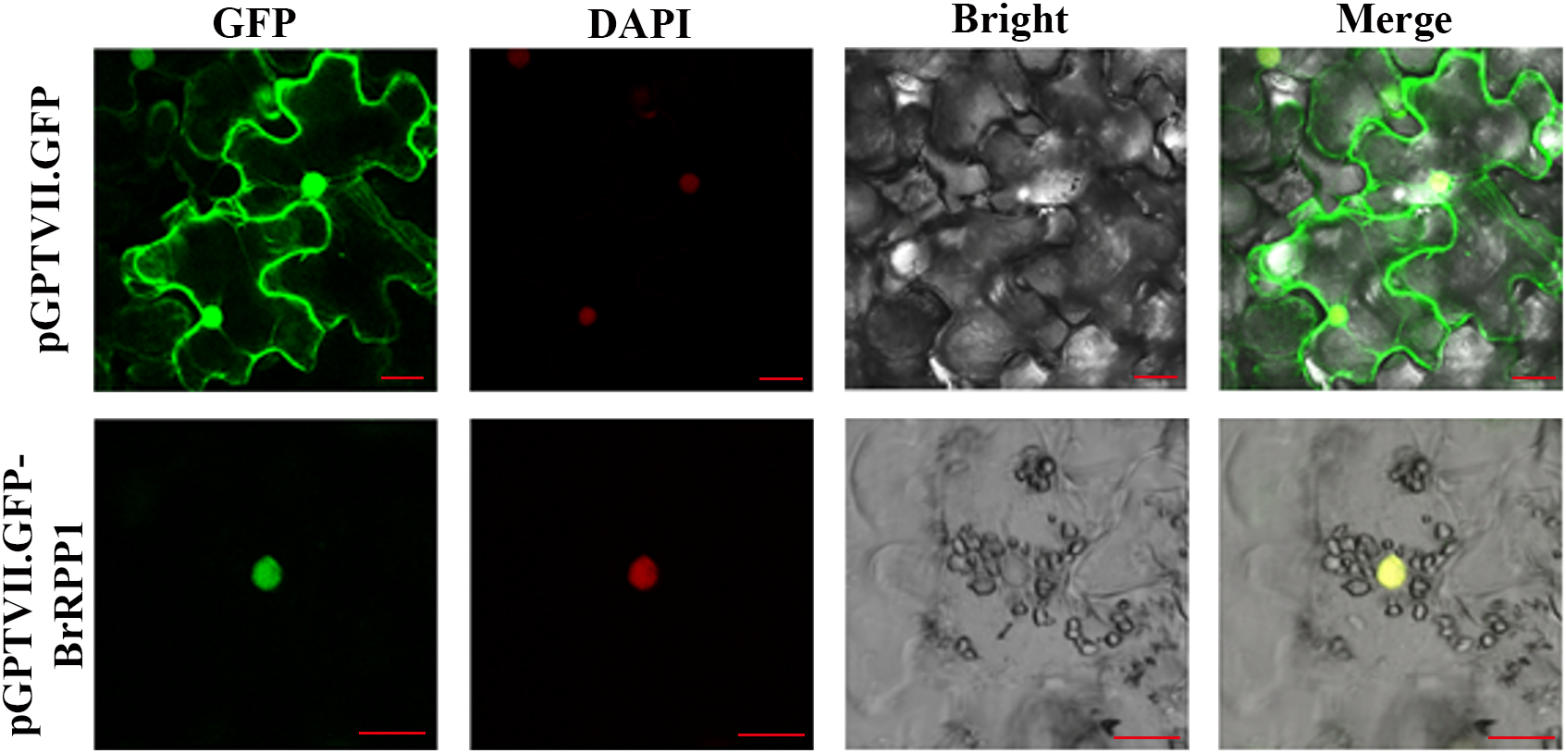
Subcellular localization of BrRPP1. GFP, GFP fluorescence. DAPI, DAPI fluorescence. Bright, brightfield image. Merge, merged pictures of GFP, bright, and DAPI fluorescence. Three views were selected for electron microscope observation. Scale bar=20 μm.

After *A. tumefaciens* infection solution containing pGPTVII.GFP-*BrRPP1* plasmid was injected into tobacco leaves, observation under Confocal laser microscope found that pGPTVII.GFP-*BrRPP1* carrier only produced fluorescence signal in the nucleus, and the fluorescence signal overlapped with DAPI nuclear dye (**Figure 5**).

### 3.6 Screening of interaction proteins of *BrRPP1*

**Figure 6:**
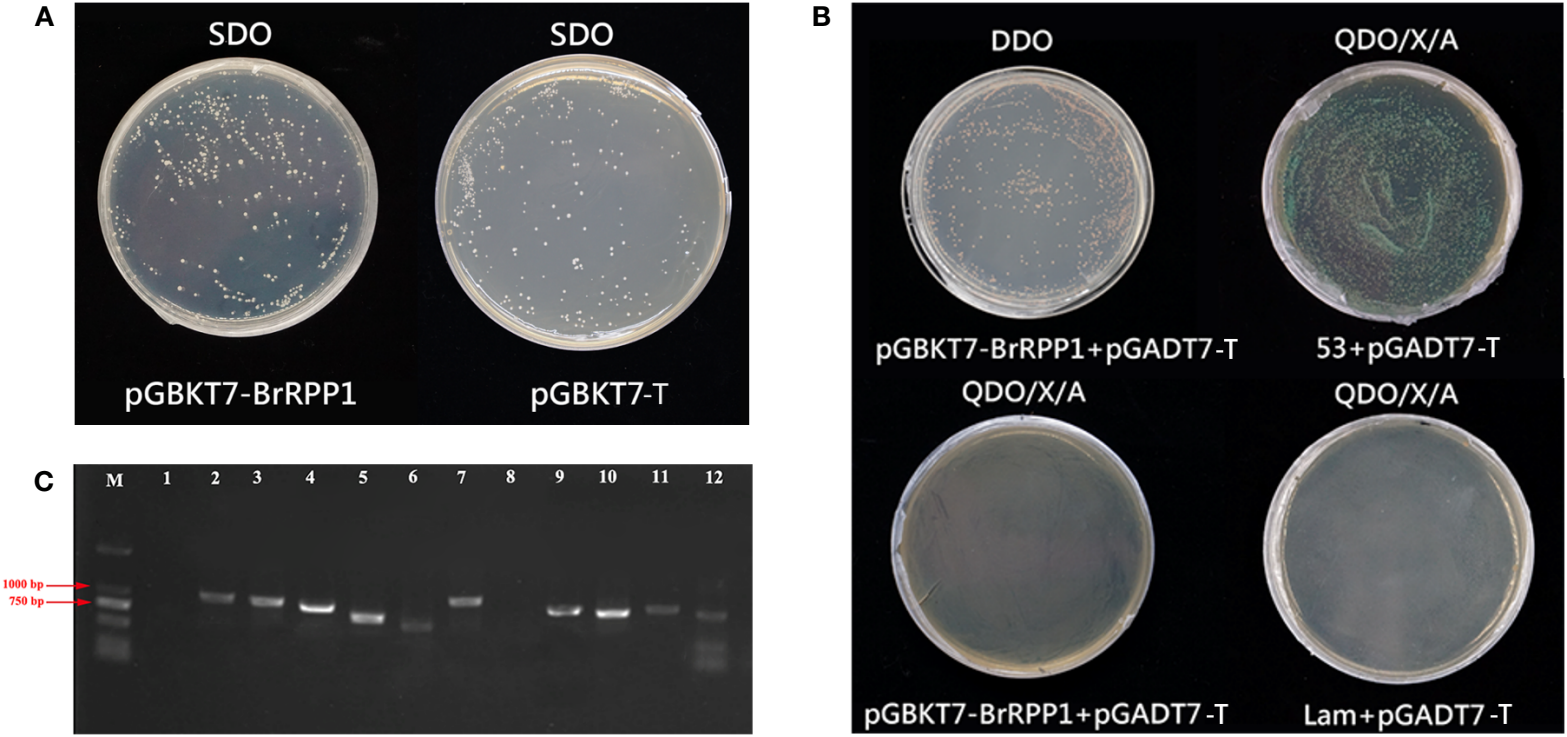
Interaction proteins screening of pGBKT7-BrRPP1. **(A)** Toxicity test of pGBKT7- BrRPP1. **(B)** Self-activation detection of pGBKT7- BrRPP1. **(C)** PCR identification of positive interaction proteins of BrRPP1 (part).

The toxicity test results of pGBKT7-BrRPP1 showed that the yeast strains containing pGBKT7-BrRPP1 could grow normally on SD/-Trp medium plate, and there was no significant difference in colony size and number between the strains containing pGBKT7-BrRPP1 and those containing pGBKT7-T, which indicated that BrRPP1 is not toxic to yeast growth (**Figure 6A**).

To avoid the false positive interaction proteins caused by the self-activation of pGBKT7-BrRPP1, self-activation analysis found that the yeast colonies co-transfected with pGBKT7-BrRPP1 and pGADT7-T could grow normally on DDO deficient medium, indicating that pGBKT7-BrRPP1 and pGADT7-T were successfully co-transfected into yeast cells. On the QDO/X/A medium, the positive control group (pGBKT7-53 + pGADT7-T) grew normally, activated the reporter genes and showed blue colonies. However, the negative control group (pGBKT7-Lam + pGADT7-T) could not grow on the QDO/X/A medium. The experimental group (pGBKT7-BrRPP1 + pGADT7-T) also could not grow on the QDO/X/A medium, indicating that the bait vector pGBKT7-BrRPP1 had no self-activation effect and could be used for yeast two-hybrid library screening (**Figure 6B**). Through Y2H library screening, 35 positive colonies were screened on QDO/X/A medium, which may interact with BrRPP1. The length of the inserted fragments of these clones ranged from 750-1000 bp (**Figure 6C**).

Functional analysis of these candidate interacting proteins shows that they participate in six pathways, including programmed cell death, JA pathway, plant cell wall synthesis, plant photosynthesis, nitrogen metabolism and transcriptional regulation. XM_009125371.3 (cytochrome c6, Cytc) involved in programmed cell death, the detection rate was 17.14%; XM_009112750.3 (Phospholipase A1, PLA1) involved in the synthesis of jasmonic acid (JA), the detection rate was 14.28%; XM_013801607.2 (galacturonosyltransferase 8-like, GT8) involved in cell wall modification, the detection rate was 11.42%; XM_009151660.3 (HNH endonuclease, HNH), XM_018654018.2 (phosphoribulokinase, PRK) and XM_009132313.1 (oxygen enhancing protein, OEE) involved in photosynthesis, the detection rate were 17.14%, 14.28% and 11.42% respectively. The detection rate of other candidate proteins is lower than 3%. In addition, there were two other unknown functional proteins (**Table 3**).

**Table 3:** Sequencing analysis of positive clones.

Accession NO.	Functional annotation	Related pathway	Detection rate
XM_009125371.3	cytochrome c6, chloroplastic, (Cytc)	programmed cell death	17.14%
XM_009112750.3	phospholipase A1-IIalpha-like, (PLA1)	JA pathway	14.28%
XM_013801607.2	galacturonosyltransferase 8-like, (GT8)	synthesis of plant cell wall	11.42%
XM_009151660.3	HNH endonuclease, (HNH)	plant photosynthesis	17.14%
XM_009132313.1	oxygen-evolving enhancer protein 1-1 (OEE)		14.28%
XM_018654018.2	phosphoribulokinase, chloroplastic (PRK)		11.42%
XM_013894031.2	glutamine synthetase cytosolic isozyme 1-3-like	Nitrogen Metabolism	2.85%
XM_009113886.3	CHCH domain protein	Transcriptional regulation	2.85%
XM_009130439.3	transcription initiation factor IIE subunit beta		2.85%
XM_013864966.2	ubiquitin-associated protein	Unknown	2.85%
XM_009118040.3	*Brassica rapa* CTD small phosphatase-like protein		2.85%

### 3.7 Analysis of relative expression of related proteins

**Figure 7:**
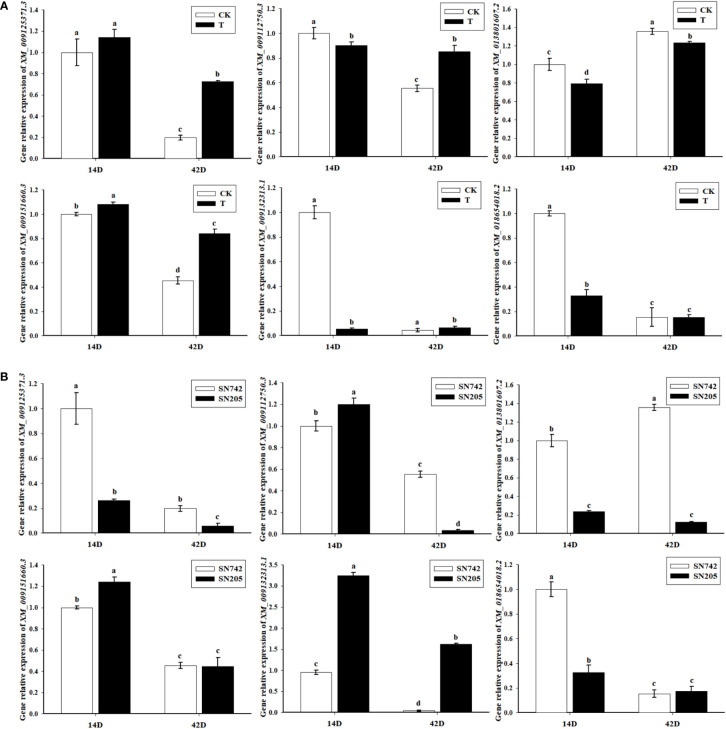
Gene expression pattern analysis of the candidate interacting protein of BrRPP1. **(A)** The expression difference of the candidate interacting proteins of *BrRPP1* between uninoculated and inoculated roots with *P. brassicae.* 14D, the 14th day after inoculation of *P. brassicae*. 42D, the 42th day after inoculation of *P. brassicae*. CK, uninoculated control roots. T, roots treated with *P. brassicae.* **(B)** The expression of the candidate interacting proteins of *BrRPP1* in roots of ‘SN742’ and ‘SN205’ uninoculated with *P. brassicae*. Data represent the mean ± standard deviation (n = 3); letters indicate significant differences at p ≤ 0.05, according to Duncan’s multiple range test.

In order to further analyze whether the expression of these candidate interacting proteins were influenced by *P. brassicae* infection, qRT-PCR results showed that the gene expression of all of these proteins were significantly changed after infection with *P. brassicae*. After inoculation treatment by *P. brassicae*, XM_009132313.1 and XM_018654018.2 were only significantly down-regulated expressed at the early stage of infection, and the expression of XM_009125371.3 and XM_009151660.3 were significantly up-regulated at both sampling dates, and XM_013801607.2 was significantly down-regulated at both sampling dates, while the expression of XM_009112750.3 was significantly down-regulated on the 14^th^ day, and significantly up-regulated on the 42^nd^ day (**Figure 7A**). In addition, expression differences of these genes were also happened between ‘SN205’ and ‘SN742’ (**Figure 7B**).

## 4 Discussion

### 4.1 BrRPP1 belongs to R gene with TIR-NBS-LRR domain

The clubroot disease caused by the *P. brassicae* has seriously affected the economic value of cruciferous crops. At present, many *R* genes to clubroot disease have been mapped in Chinese cabbage. As a common candidate gene for *Rcr1* and *Rcr2* (Chu et al., 2014; Yu et al., 2016; Huang et al., 2017), *Bra019410* was a highly homology protein with *RPP1* in *Arabidopsis*, so it was named *BrRPP1*. Through the analysis of the conservative domain, we found that the protein contains TIR and LRR and other conservative domains, belonging to TIR-NBS-LRR proteins (**Figure 1A**). The LRR domain of TIR-NBS-LRR has the function of identifying pathogens and can trigger the ETI resistance of plants (Ghelder and Esmenjaud, 2016). Previous studies have confirmed the LRR domain of *RPP1*-NdA (allele of *RPP1*) of hyaloperospora *Arabidopsidis* can recognize the effector protein ATR1 of the *oomycete* pathogen, but the LRR domain alone cannot activate the hypersensitivity of plants, which indicates that other domains of *RPP1* have signal transduction functions (Steinbrenner, 2015).

### 4.2 Sequence difference of *BrRPP1* between resistant and susceptible materials leads to resistance difference to *P. brassicae*

After cloning and sequencing of cDNA sequence of *BrRPP1* in ‘SN205’ and ‘SN742’, it was found that the cDNA sequence was different between ‘SN205’ and ‘SN742’, and the difference mainly occured after the LRR conservative domain (**Figure 1**). In addition, sequence and elements number of *BrRPP1* promoter were significantly differences between resistant and susceptible materials (**Figure 2**). The analysis of gene expression pattern showed that the expression of *BrRPP1* was not affected by the infection of *P. brassicae*, while the expression of *BrRPP1* in ‘SN205’ was significantly higher than that in ‘SN742’ (**Figure 3**). Therefore, it can be inferred that the sequence and the expression difference of *BrRPP1* leads to resistance difference between resistant and susceptible materials.

### 4.3 *BrRPP1* may play a role in disease resistance to *P. brassicae*

*Arabidopsis thaliana* can also be infected by *P. brassicae*, so it can be used as a useful model host for studying the clubroot disease (Koch et al., 1991; Mithen and Magrath, 1992; Ludwig-Müller et al., 2009). In order to confirm the disease resistance of *BrRPP1*, the disease resistance of the *Arabidopsis* mutant *rpp1* was identified. The results showed that the deficiency of *RPP1* accelerated the infection of *P. brassicae*, which indicated that *BrRPP1* should play a role in disease resistance to *P. brassicae* (**Figure 4**).

### 4.4 *BrRPP1* was expressed in the nucleus

In order to analyze the expression site of *BrRPP1* in cells, we constructed the expression vector pGPTVII.GFP-*BrRPP1* for subcellular localization analysis. In previous subcellular localization studies, the *RPP1*-WsA fragment was located in the endoplasmic reticulum and Golgi apparatus, *RPP1*-WsB fragments are located in the plasma membrane, and *RPP4* and *RPP8* fragments are located in the nucleus (Takemoto et al., 2012). However, the localization result of the N-terminal fragment alone cannot completely determine the expression pattern of the full-length protein. GhTIR-NBS-LRR1 containing the structure of TIR-NBS-LRR was a homologous protein of *BrRPP1*, which localized in the nucleus (Li et al., 2019). This result provided a reference for our prediction results. In this experiment, Confocal laser microscope observation showed that pGPTVII.GFP-BrRPP1 appeared fluorescence signal in the nucleus, which confirmed *BrRPP1* was expressed in the nucleus (**Figure 5**). Therefore, yeast two-hybrid screening can be carried out using nuclear library.

### 4.5 *BrRPP1* may regulate programmed cell death by promoting the release of cytochromes in response to be infection of *P. brassicae*

The *RPP1* (Recognition of Peronospora parasitica1) was first found in *Arabidopsis* ‘*Wassilewskija*’ (Botella, 1998). The current research about *RPP1* is mostly in its interaction with effector proteins, intramolecular interactions and the process of inducing plant cell death. The *Arabidopsis RPP1* is a typical TIR-NBS-LRR gene, *RPP1* has been shown to be able to directly recognize pathogens in *Arabidopsis thaliana*, and it also has an indirect recognition function by recognizing the target proteins of pathogenic effector proteins (Renier et al., 2008). TIR domain in *RPP1* has been confirmed to have a self-binding effect, which plays an important role in inducing cell death (Wan et al., 2019). In this study, 35 interacting proteins of BrRPP1 were screened by yeast-two hybrid. Among them, Cytochrome C (Cytc) is widely present in the mitochondria of plants and organisms, and is considered to be related to the programmed cell death. The release of Cytc from mitochondria is common in the process of programmed cell death in plant cells (Vacca et al., 2006; Qi et al., 2018; Matilla, 2021). Wang et al. (2016) also found that rice NBS-LRR disease-resistant protein Pik-h interacts with Cytochrome c oxidase assembly protein (COX11). In our study, it was found that the expression of *Cytc* was significantly up-regulated after infection by *P. brassicae*, and its expression was significantly different between resistant and susceptible materials (**Figure 7**). Therefore, we can speculate that *BrRPP1* as a TIR-NBS-LRR gene may regulate the programmed death of plant cells by promoting the release of cytochromes.

### 4.6 BrRPP1 may interact with BrPLA1 to mediate JA pathway in response to infection of *P. brassicae*

Jasmonic acid (JA) defense response is necessary for defense against biotic stress (Wasternack, 2007). *AtPLAs* family is involved in the transduction of auxin and pathogen signals (Holk et al., 2002). *PLA2* can be strongly induced when tobacco is infected by bacterium *Erwinia carovora* or the fungus *Botrytis cinerea*, and accumulate large amounts of JA and 12-oxo-phytodienoic acid (Dhondt et al., 2010). We also found that a lipoyl hydrolase phospholipase A1 (PLA1) interacts with BrRPP1, which is homologous to *Arabidopsis AtPLAIIα* (**Table 3**).

There is a conflict between the understanding of relationship of JA pathway and the infection of *P. brassicae*. Eight genes related to JA biosynthesis and signaling were down-regulated in *Brassica rapa* ‘CR BJN3-2’ at early defense response induced by *P. brassicae* infection (Chen et al., 2016). Jubault et al. (2013) also reported the occurrence of a suppressed JA/ET signaling pathway in *Arabidopsis* on the 7^th^ day after infection by *P. brassicae.* However, in the transcriptome experiment of Luo et al. (2018), the JA pathway was significantly up-regulated after infection by *P. brassicae*. Lemarié et al. (2015) demonstrated that both the SA and JA pathways contribute to resistance against *P. brassicae* in *Arabidopsis*.

In our research, the relative expression of *PLA1* was significantly down-regulated on the 14^th^ day after infection by *P. brassicae*, while the expression was significantly up-regulated on the 42nd day after infection, and its expression was significantly different between resistant and susceptible materials (**Figure 7**). Therefore, we speculate that BrRPP1 may interact with PLA1 to mediate the regulation of JA, but the process of JA pathway is complex in response to infection of *P. brassicae*. The confliction of JA pathway responsing to infection of *P. brassicae* should be from the different detection stage after infecting by *P. brassicae*.

### 4.7 *BrRPP1* may modify the plant cell wall in response to the infection of *P. brassicae*

In addition, one of the screened interacting proteins of BrRPP1, XM_013801607.2, belongs to the glycosyltransferase family 8 (GT8), which has high homology with *Arabidopsis AtGAUT8* (At3g25140). It has been proved that alpha-galacturonosyltransferase (GAUT) can synthesize pectin with UDP-GalA as substrate to participate in the synthesis of plant cell wall (Sterling et al., 2006). Plant cell walls provide a structural framework to support plant growth and act as the first line of defense when plants encounter pathogens (Houston et al., 2016). The changes of cell wall components affect the downstream functions of cells as storage units, structural networks and solute transporters. In many cases, it also affects the ability of cells to respond to stress caused by pathogens and the environment (Tucker et al., 2018). Genes encoding enzymes that can synthesize or hydrolyze plant cell wall components show different expressions under different pressures, indicating that they may promote stress tolerance by changing cell wall components (Seki et al., 2002). The arabinogalactan protein (Unigenes40439), a cell wall component, was changed significantly after infection by *P. brassicae* (Luo et al., 2018). In this study, the relative expression of GT8 was significantly down-regulated after infection by *P. brassicae*, and its expression was significantly different between resistant and susceptible materials (**Figure 7**). Therefore, it can be speculated that BrRPP1 may modify the plant cell wall to respond to the infection of *P. brassicae* by regulating glycosyltransferase.

### 4.8 BrRPP1 may interact with photosynthetic pathway proteins to mediate the response of Chinese cabbage to be infected by *P. brassicae*

Among these interacting proteins, some proteins related to plant photosynthesis have been found, including oxygen-evolving enhancer protein (OEE), phosphoribulokinase (PRK) and His-Asn-His endonuclease (HNH) (**Table 3**).

OEE is the peripheral protein of photosystem II and plays an important role in the oxygen releasing activity of photosystem II (Seidler et al., 1996). Studies have found that *Arabidopsis* oxygen enhanced protein *AtOEE2* may participate in the generation of reactive oxygen species in the ETI reaction of plants by acting as the downstream signal of *WAK1* (Yang et al., 2003). Oxygen enhancing protein has been proved to be involved in the transduction of disease resistance signals. The *Phytophthora capsici* Effect Factor RxLR19781 can regulate its zoospore infection by combining with the oxygen enhancing NbOEE2, which is the target protein of the effector protein of pathogenic bacteria (Liang et al., 2019). In this study, it was found that the expression of *OEE* was significantly down-regulated on the 14^th^ day after inoculation of *P. brassicae*, and its expression level in resistant and susceptible materials was significantly different (**Figure 7**), so we speculated that OEE, as a peripheral protein of photosystem II, may be the target protein of effector protein of *P. brassicae*, and participate in the indirect recognition of BrRPP1 to *P. brassicae*.

The PRK is a part of the Calvin cycle of plant dark reaction, which exists in chloroplasts, and is responsible for the fixation of CO_2_ in photosynthetic organisms by catalyzing ribulose 5-phosphate to form the CO_2_ receptor ribulose 1, 5-diphosphate (Hariharan and Cattolico, 1998). Although people usually focus on the role of PRK in carbon assimilation and in the light environment, PRK also plays a role in biological and abiotic stresses. For example, *OsPRK* gene may participate in the induced defense response of rice to pests (Chen et al., 2020), and *PRK* in wheat responded to the infection of *Puccina striiformis* (Liang et al., 2007). In this study, it was found that the expression of *PRK* was significantly down-regulated after inoculation with *P. brassicae*, and its expression was significantly different between resistant and susceptible materials (**Figure 7**). This suggests that PRK, as a part of the Calvin cycle of plant dark reaction, may respond to the infection of *P. brassicae*.by interacting with BrRPP1,

The HNH nuclease domain contains three of the most conserved His and Asn amino acid residues and has DNA cleaving activity (Zhang et al., 2016). The role of proteins containing HNH nuclease motif has been rarely reported in plants. A recent study showed that a White Stripe Leaf 9 (WSL9) protein containing HNH domain plays a crucial role in the early development of chloroplasts (Zhu et al., 2020). There is no report on the role of HNH in disease resistance. In this study, it was found that HNH interacted with BrRPP1, and its expression was significantly down-regulated after being infected by *P. brassicae*. The expression of HNN was significantly different between resistant and susceptible materials (**Figure 7**). This result suggests that HNH plays a crucial role in the early development of chloroplasts, and may respond to the infection of *P. brassicae*.

Above all, OEE, PRK and HNH all participate in plant photosynthesis, and respond to the infection of *P. brassicae* by interacting with BrRPP1.

## 5 Conclusions

**Figure 8:**
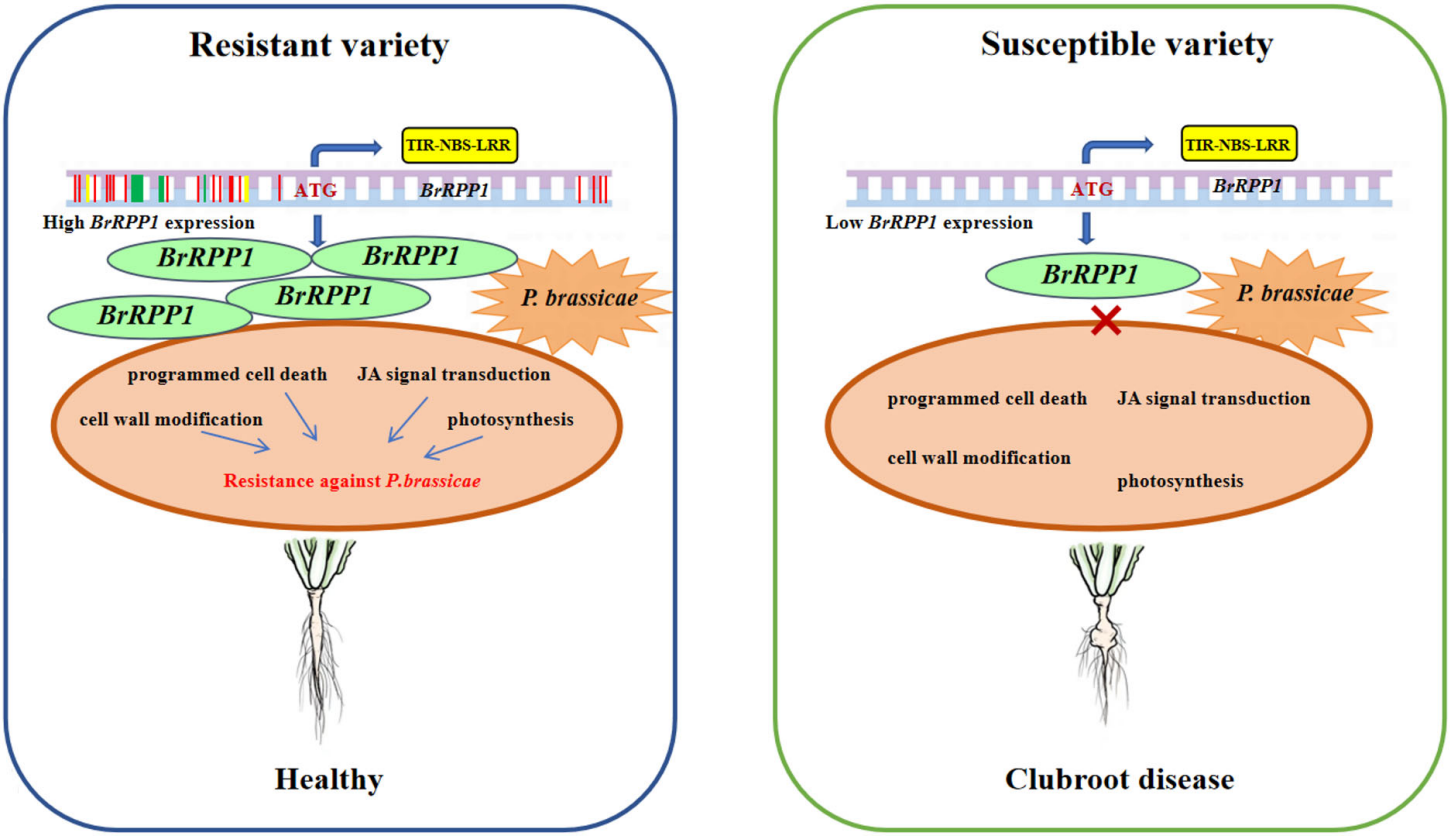
Role pattern of *BrRPP1* in Chinese cabbage response to *Plasmodiophora brassicae*. *BrRPP1* gene contains TIR-NBS-LRR domain and belongs to *R* gene. The gene sequence of *BrRPP1* in resistant varieties was different from that in susceptible varieties. Green represents base insertion, yellow represents base deletion, and red represents base mutation. The difference of *BrRPP1* promoter sequence leads to the significant difference of gene expression between resistant varieties and susceptible varieties.

*BrRPP1* has been located as a candidate *R* gene to clubroot disease for many times. In this study, we found that *BrRPP1* contains typical TIR-NBS-LRR domain of *R* gene. Although the gene expression of *BrRPP1* was not affected by the infection of *P. brassicae*, the sequence difference of its cDNA and promoter between resistant and susceptible materials leads to the change of protein structure, and the significant difference of *BrRPP1* gene expression between resistant materials and susceptible materials. *Arabidopsis* homologous deletion mutant of *BrRPP1* reduced the resistance to clubroot disease. Moreover, the interaction proteins of BrRPP1 with high detection rate were involved in the pathways of programmed cell death, JA signal, cell wall synthesis and photosynthetic, and their expressions were significantly changed by infection with *P. brassicae*. So it was speculate that *BrRPP1* participated in the resistance of Chinese cabbage to *P. brassicae* by inducing the above pathways (**Figure 8**).

## Data availability statement

The original contributions presented in the study are included in the article/**Supplementary Material**. Further inquiries can be directed to the corresponding author.

## Author contributions

WG, ML and RJ defined the research theme and wrote the manuscript. HF, XW and BZ designed methods and experiments, carried out the laboratory experiments, analyzed the data, and interpreted the results. KL, JZh, and JZo co-designed the experiments and discussed the analyses and interpretation. All authors contributed to the article and approved the submitted version.
