# Supplementary material for: Analysis of the role of BrRPP1 gene in Chinese cabbage infected by Plasmodiophora brassicae
Source: Front Plant Sci. 2023 Jan 25;14:1082395. doi: 10.3389/fpls.2023.1082395 (PMC9905630; doi:10.3389/fpls.2023.1082395)
Supplement: Supplementary file 1 [file Table_1.docx]

**Table S1. Primers used in qRT-PCR**

| **Name** | **Sequence (5’-3’)** |
| --- | --- |
| ***BrRPP1*** | Forward TGAAGTGAGGATGATAGGGA |
|  | Reverse TAGTAAGGTCTGGGAAAACG |
| ***BrActin*** | Forward ATCTACGAGGGTTATGCT |
|  | Reverse CCACTGAGGACGATGTTT |
| **HNH** | Forward TCTTCTTCTCCTTCCCCGCC |
|  | Reverse CATTGCTGCTTCACGCTGTA |
| **Cytc** | Forward CTCCTGCCTCTGCTCGCTGC |
|  | Reverse AACCGCAAGAATAACCGCCG |
| **GT8** | Forward AACTCTTCGTATGTTCCCGT |
|  | Reverse AGGCAAATAAAACCTCAAGT |
| **OEE** | Forward GCTAAAATCTCCACCGCTCC |
|  | Reverse GACAGCGTCGGAGCATTTAC |
| **PLA1** | Forward TGCGAGAATACTTCCAACAA |
|  | Reverse AAACCAGTCTACACCAACAG |
| **PRK** | Forward CGTCGTGATCGGACTCGCTG |
|  | Reverse GGGTTCCCTCCTTTTGGTGG |
